# Dr. Barnardo's Homes

**Published:** 1904-09-10

**Authors:** 


### DR. BARNARDO'S HOMES.

The August number of the *National Waifs' Magazine* contains the Annual Report for 1903 of the National Waifs' Association, and it is satisfactory to observe that the progress of this charity is one of continued prosperity. The year 1903 was the thirty-eighth in the history of the association, and so rapid has been its growth in the comparatively short time since the charity was founded that its income for the year under consideration amounted to no less a sum than £179,740. And with regard to this total it is interesting to notice that it represents the contributions of 83,576 givers, over 66 per cent. of the donations consisting of amounts under £1; and it is clear that the institution depends for its income, to a large extent, on small contributions, a fact which probably accounts in great measure for its success, and which promises well for the future. On January 1st, 1903, there were 6,399 children in the Homes; and in the course of the following twelve months there were 4,078 fresh admissions, making a total of 10,477 children under the care of the association, while many thousands of others who made application for relief and who were found not to be absolutely destitute, and therefore not admissible to the Homes, were lodged and fed for a short while, or helped with clothing, boots, and other accessories. In the course of the year 836 boys and 401 girls were emigrated to Canada, and situations were found at home for 1,631. During the year there were 64 deaths, a mortality of 6 10 per 1,000 in residence. Of the 7,078 children under the care of the association on December 31st, 1903, 3,291 were boarded out in rural cottage homes. The Report states that "the advantages or this system of boarding out, as against even the best institutional life, can hardly be over-estimated." Among the special requirements set forth at the end of the report we note the sum of £12,000 for the erection of a hospital for the Girls' Village Home at Barkingside.

